# Genomic findings with familial implications: agenda setting in light of mainstreaming

**DOI:** 10.12688/openreseurope.19128.1

**Published:** 2025-01-10

**Authors:** Amicia Phillips, Eva Van Steijvoort, Maria Siermann, Janneke M.L. Kuiper, Álvaro Mendes, Sandrine de Montgolfier, Helle Vendel Petersen, Anna Rosén, Hilde Van Esch, Laurent Pasquier, Danya F. Vears, Christine Patch, Wannes Van Hoof, Ainsley J. Newson, Saskia Bulk, Carla van El, Eline Dancet, Emmanuelle Rial-Sebbag, Colin Mitchell, Pascal Borry

**Affiliations:** 1 Department of Public Health and Primary Care, Centre for Biomedical Ethics and Law KU Leuven, Leuven, Flanders, 3000, Belgium; 2 Department of Clinical and Biomedical Sciences, University of Exeter Medical School, Exeter, England, UK; 3 Department of Health, Ethics & Society, Maastricht University GROW School for Oncology and Reproduction, Maastricht, Limburg, The Netherlands; 4 Department of Sociology, Centre for Sociological Research KU Leuven, Leuven, Flanders, 3000, Belgium; 5 Centre for Predictive and Preventive Genetics, IBMC – Institute for Molecular and Cell Biology, University of Porto, Porto, Portugal; 6 i3S – Instituto de Investigação e Inovação em Saúde, University of Porto, Porto, Portugal; 7 Aix Marseille Univ, Inserm, IRD, SESSTIM, Sciences Economiques & Sociales de la Santé & Traitement de l’Information Médicale, ISSPAM, Marseille, France; 8 Université Paris Est Créteil, Créteil, France; 9 Medical department, Zealand University hospital, Nykøbing Falster, Denmark; 10 Department of people and Technology, Roskilde University, Roskilde, Denmark; 11 Department of Diagnostics and Intervention, Unit of oncology, Umeå University, Umeå, Sweden; 12 Centre for Human Genetics, University Hospitals Leuven, Leuven, Belgium; 13 Clinical genetics, Reference Center for Rares Diseases “Intellectual Disabilities”, Rennes University Hospital, Rennes, France; 14 Murdoch Children’s Research Institute, The Royal Children’s Hospital, Parkville, Australia; 15 Department of Paediatrics, University of Melbourne, Parkville, Australia; 16 Wellcome Connecting Science, Hinxton, England, UK; 17 Cancer center Sciensano, Brussels, Belgium; 18 Sydney Health Ethics, Sydney School of Public Health, Faculty of Medicine and Health, The University of Sydney, Sydney, Australia; 19 Service de Génétique Humaine, CHU de Liège, Liège, 4000, Belgium; 20 Department of Human Genetics, Section Community Genetics, Amsterdam UMC, Vrije Universiteit Amsterdam, Amsterdam, The Netherlands; 21 Amsterdam Public Health Research Institute, Amsterdam, The Netherlands; 22 CERPOP, UMR 1295, BIOETHICS Team, INSERM, University of Toulouse, Toulouse, France; 23 PHG Foundation, University of Cambridge, Cambridge, UK

**Keywords:** Family communication, personalized prevention, genetic risk, genetic screening, cascade screening, policy, genetic counseling

## Abstract

An international workshop was held in Leuven, Belgium, on June 19–20, 2023, to discuss the communication of genetic risk information within families in the context of personalized prevention. Organized as part of the Horizon Europe project PROPHET (PeRsOnalised Prevention roadmap for the future HEalThcare in Europe), the event gathered interdisciplinary stakeholders to explore the benefits and challenges of various policy approaches for returning genetic test results with implications for family members. Five key themes emerged from the discussions: (1) recognizing family communication as an ongoing process, (2) adopting a family-centered approach rather than an individual one, (3) clarifying roles and responsibilities in the communication process, (4) addressing the lack of clear guidelines and policies, and (5) ensuring sufficient resources. To enhance family communication of genetic risk information, participants emphasized the importance of improving pre-test counseling and follow-up procedures, implementing policies to clarify roles and responsibilities, and providing training for healthcare professionals both within and outside genetic services.

## Plain Language Summary

An international workshop took place in Leuven, Belgium, on June 19–20, 2023, to discuss how to share genetic risk information with families as part of personalized prevention. The event was organized through the Horizon Europe project PROPHET, which aims to shape the future of healthcare in Europe. The workshop brought together people from different fields to look at the challenges and benefits of sharing genetic test results that could affect family members. Five main ideas came out of the discussions: (1) treating family communication as an ongoing conversation, (2) focusing on the family as a whole rather than just the individual, (3) making roles and responsibilities in the communication process clearer, (4) addressing the lack of clear rules and policies, and (5) ensuring there are enough resources to support these efforts. To improve how genetic risk information is shared with families, the participants stressed the importance of better counseling before genetic tests, clearer follow-up steps, clearer policies, and training for healthcare workers both within and outside genetic services.

## Introduction

Genomic sequencing is being increasingly implemented into mainstream clinical practice. Findings, which can sometimes be unexpected, can have implications not only for individuals, but also for their relatives. And while genetic information may be considered personal, it is also inherently familial ^1^. Identification or confirmation of a hereditary condition in an individual (whether by DNA analysis or another method) can indicate that other family members may develop the condition and could also pass it to any future children. When a heritable genetic condition is present in a family, disseminating that information to family members may help connect relatives with counseling, testing, prevention, treatment, and reproductive options.

Although the familial communication of genetic information is widely seen as important, discussing genetic risk in a family can cause distress. Some patients struggle to initiate these conversations with their family members ^2^. Conversations can be challenging, and patients may worry that disclosing information about genetic risk could be harmful to their relatives. It can be difficult for patients to find the “right time” to broach the subject ^3^. In some cases, patients may feel guilty about the genetic diagnosis. Furthermore, patients may feel overly burdened with the responsibility to inform their relatives, especially considering that some may be coping with their own diagnosis and treatment at the time. All these concerns are further compounded when patients have distant or strained relationships with their relatives ^4– 8^. So, despite the fact that most patients have the intention to inform their at-risk relatives and cases of active nondisclosure are rare (where patients explicitly object to disclosure), empirical research indicatess that this communication often does not take place in practice ^9– 12^.

While the responsibility to inform at-risk relatives is by default the responsibility of the index patient, due to the challenges of family-mediated contact, alternative models of informing relatives are being explored whereby healthcare professionals or the healthcare system inform relatives. Several countries have trialled or implemented these different models of direct contact (with the patient’s consent), but research indicates varying effects on family members being contacted in this way ^13^ and degrees of impact on uptake of genetic testing/cascade testing, although this is admittedly an imperfect measure of family communication ^14, 15^. Knowing the challenges that patients might face with familial communication of genetic risk, some genetics services offer additional support for patients in the process ^16^, although practices vary significantly between healthcare settings and individual healthcare professionals. Genetic counseling may be provided to patients who need further support with informing their relatives, although its availability varies significantly between countries ^17– 19^. Genetics services may also in cases of actionable genetic conditions provide a ‘family letter’ which informs the recipients about their genetic risk and encourages them to seek testing ^20^. Usually, the patient is given the letter and asked to distribute it to relevant family members. In some cases, with the patient’s consent, the healthcare professional may distribute it on their behalf.

Despite the availability of support to assist family communication (although it should also be noted that substantial heterogeneity exists between contexts), a proportion of patients still do not communicate genetic risk information to family members. In this instance, relatives who could benefit from knowledge of a genetic risk in the family are left uninformed, and without the option to make their own decisions about potential testing and prevention. While most patients intend to communicate and rarely actively object to disclosure, when they do, the patient’s right to confidentiality and autonomy comes in conflict with the interests of the relatives who could benefit from knowledge of a genetic risk in the family. In cases where the healthcare professional is aware of nondisclosure, they are confronted with the dilemma of how to balance these two conflicting sides; while they have a duty to care for their patient and a duty to protect their confidentiality, they may also feel responsible, or might in fact be ethically and in some jurisdictions legally responsible, for preventing harm for the patient’s at-risk relatives ^21– 23^. While not common, during their careers many healthcare professionals encounter this dilemma, and yet they often feel that they lack the proper resources to help them make a decision ^5, 22^.

Although the patient has a legal right to confidentiality, this is not absolute, with exceptions already being widely accepted in the context of infectious diseases or when a patient poses a serious and imminent threat to themselves or others. The question arises whether, in the context of genomics, a breach in confidentiality can ever be warranted and if so, under which conditions. Alternatively, some authors sidestep this dilemma by positing that disclosing genetic risk information to family members does not necessarily violate the patient’s confidentiality ^24– 26^. The crux of this argument rests upon the fact that while in some ways genetic information is personal, it is inherently familial and shared. This means that confidentiality can be considered at a familial level whereby genetic information belongs to both the individual patient and their family. Informing relatives of a genetic risk in the family thus does not breach the patient’s confidentiality because as Lucassen and Gilbar (2018) argue “no identifiable information is communicated in such a statement, even if genetic findings in one person first led to that conclusion” ^25^.

To add a further layer of complexity to the ethical challenge of cases of nondisclosure, there is also the relative’s right to know and right not to know information about their own genetic risk. On the one hand, relatives may have a legal right to information that pertains to their health and that could prevent harm ^27^. On the other hand, relatives may have a right not to know information that they might find distressing or irrelevant either clinically or regarding their everyday life ^28^. Notably, some argue that informing someone that they are at risk does not necessarily violate the right not to know since the person informed can still choose whether they want to get tested ^29^, but this argument remains controversial as some believe knowing one has a heightened risk still violates their right not to know. In several jurisdictions the right to know and the right not to know are not only ethical rights, but also have a legal backing ^27^. Whoever is disclosing the information, whether it is the patient or the healthcare professional, must consider these possibly competing rights when making decisions about disclosure.

Several countries have policies that clarify the ethically complexities of communicating genetic risk in families. In some countries, this is through the form of policies that have been developed specifically to address the issue of family communication. The approaches taken differ significantly. In Australia, for example, regulations made under Federal legislation (and mirrored in some states) have made it possible for some healthcare professionals to breach confidentiality to inform family members in some circumstances ^30– 34^. In the UK, the court case ABC v St Georges Healthcare NHS Trust & Ors asserts that genetic healthcare professionals have a legal duty to weigh the interests of patients and their at-risk relatives, meaning in some exceptional cases a healthcare professional could be obliged to inform relatives of their genetic risk ^35^. In the US, healthcare professionals are neither required nor permitted to disclose without patient consent under the Health Insurance Portability and Accountability Act (HIPAA) Privacy Rule ^36^. In France, a law has imposed a duty on the patient to inform family members either directly or indirectly via their healthcare professional ^37^. More commonly, as is the case in Belgium, no specific guideline or law exists that addresses the rights and responsibilities of patients, healthcare professionals, and at-risk relatives, meaning it is unclear what can be done in cases of nondisclosure ^38^.

As genomics becomes increasingly integrated into mainstream healthcare, findings with significant familial implications are more frequently encountered in clinical practice. The broad scope of genomic sequencing introduces new layers of complexity and with that concerns due to the significant variation in the degree to which the causes, penetrance, and expression of genetic conditions are understood. Furthermore, with genomics moving beyond specialty settings, such cases are now often handled by healthcare professionals with limited experience in genetics and genomics, and patients may be unprepared for the responsibility of informing family members. As genomic applications continue to expand in both research, clinical, and public health contexts, it is essential to revisit and re-evaluate how genetic risk information is communicated within families, addressing both ethical and practical considerations.

### Organization of an international workshop

On June 19th and 20th 2023, a workshop was held in Leuven, Belgium with an expert panel consisting of interdisciplinary stakeholders involved in the area of family communication of genetic risk information either through research, clinical practice, or policy. Participants came from eight countries, namely Belgium, the Netherlands, France, the UK, Australia, Denmark, Sweden, and Portugal. Organized in the context of the Horizon Europe project PROPHET (PeRsOnalised Prevention roadmap for the future HEalThcare in Europe), the workshop aimed to support the development of guidance for returning genetic test results with familial consequences in the context of personalized prevention ^39^. One of the main motivations of organizing the workshop on communicating genetic risk in families was to foster debate on the benefits and concerns of different policy approaches. The workshop consisted of a general overview of the ethical issues involved in family communication, as well as presentations on specific policy approaches adopted by France, Australia, the UK, and Denmark. These concrete examples provided reference points for the ensuing in-depth discussions on the roles and responsibilities of patients and healthcare professionals, and whether for some conditions an approach drawing on public health values and practices should be applied to ensure at-risk individuals are informed. Based on the input gained during the workshop discussions, this paper provides an overview of the ethical and legal landscape as well as identifying future research priorities. From the discussions five main themes emerged that we hope will foster conversation on the topic, namely: 1) the emphasis on family communication as a process, 2) the shift to a familial approach, 3) roles and responsibilities in the communication process, 4) the need to clarify guidelines and policy, and 5) the importance of adequate resources.

***1. Emphasis on family communication as a process***

Participants emphasized how family communication is a complex, multi-step process. Participants pointed out how the language of disclosure currently used in the clinic, literature, guidelines, and policies can imply that family communication is a one-time event rather than an ongoing process in need of continual re-evaluation and revisitation. To enhance this process, several participants proposed that prior to testing, healthcare professionals and patients create an informal “contract” between both parties to clarify respective responsibilities and expectations to prevent misunderstandings following the return of results.

The complexity of the process of family communication necessitates a nuanced approach by the healthcare team to support patients along this journey, creating a shared responsibility between healthcare professionals and patients. Participants noted the importance of distinguishing between merely transmitting information and engaging in meaningful family communication, requiring healthcare professionals to encourage and support this dialogue beyond information provision and beyond the consultation where the results are returned. Participants advocated for patient involvement from the outset of genetic/genomic testing, with an emphasis on informing them about the possible consequences of genetic/genomic tests and addressing their concerns. Clear communication guidelines, including who, what, why, and when to inform, supported by communication tools, can assist both patients and healthcare professionals in navigating expectations and responsibilities throughout the process of family communication. These can then be adapted to suit the contextual features of different patient and clinical scenarios, as well as the potential utility of the information.

The importance of healthcare professionals and patients discussing familial implications and dissemination of results together throughout the sequencing timeline (i.e., during pre-test counseling, return of results, and follow up) emerged as a core theme of the workshop discussions as we discuss below in further detail.

*Pre-test counseling*

Research indicates that addressing family communication earlier in the genetic testing or genomic sequencing process improves rates of disclosure in families ^10, 40^. However, the possibility of uncovering results with familial implications is not uniformly addressed in pre-test consultations. Practices regarding the content of pre-test counseling discussions vary substantially; in some cases, familial implications are discussed prior to testing since they are the main aim of testing, but in other instances, familial implications are seen as more of a secondary outcome and thus not addressed at all pre-test. For instance, a palliative patient with colorectal cancer, might be offered genetic testing in order to help their relatives by determining whether there is a pathogenic variant in the family. This contrasts with cases where patients present with symptoms with no known cause, and thus finding a diagnosis for the patient is the primary objective of testing, meaning that familial implications may not always be addressed before testing.

While participants generally agreed that discussing family communication prior to sequencing is best practice, some pointed out how, in some contexts, adhering to this standard may prove challenging. For example, when genomic sequencing, as opposed to targeted genetic testing, is used, due to its broader scope, it is more likely to generate unexpected and uncertain findings. The difficulty with predicting findings in this context understandably complicates the clinical team’s ability to inform patients about the possibility for familial implications. Participants raised that informing patients of this potential outcome, without being able to provide further information about the kind of genetic risk that could be identified, could be potentially distressing for patients.

Bearing in mind these challenges, participants nevertheless agreed that, to the best of their abilities, healthcare professionals should discuss the potential for familial implications prior to testing to help make patients aware of this possibility and the need to inform their relatives. Furthermore, one participant noted how initiating conversations with family members prior to testing could in some cases lower the barriers for patients to eventually share their results.

*Post-test consultation*

Participants agreed that when healthcare professionals return results, it is important that they also address familial implications and the importance of disseminating information about the genetic risk within the family. Healthcare professionals should help patients to identify which family members are at-risk and could benefit from being informed. At the same time healthcare professionals should ensure that patients understand that a heightened risk is not the same as a diagnosis. While this was noted by participants to be standard practice in their respective contexts, empirical research with patients demonstrates that patients often misunderstand or do not sufficiently understand who should be informed and what to communicate to relatives ^9, 19, 20^. Participants supported current practice whereby in the post-test consultation healthcare professionals provide patients with family letters to help them disseminate information about the familial genetic risk. If it appears that a patient will not inform their relatives, participants identified connecting patients with additional counseling or psychological resources as an important strategy to support patients. Integrating counselors and psychologists in the consultations with patients (as part of the team of healthcare professionals) was identified as a strategy of improving the utilization of these services by patients. It should be noted though that patients may be struggling to cope with their own diagnosis and treatment at the time of the return of results which may introduce challenges to addressing family communication in the initial post-test consultation.

*Follow up*

Participants noted the importance of following up with patients after some time has elapsed since the initial return of results as a crucial step in ensuring disclosure. Participants working in clinical practice also noted that most patients do not outwardly object to informing their relatives. Instead, many patients intend to communicate, but delay doing so because they do not know how to broach the subject, and they may worry about causing their relatives distress ^8, 10, 41^. Patients may struggle to process that others in their family are at-risk and may even feel guilty about informing their relatives, especially if they feel responsible for passing the condition on to their children. If a patient appears to be struggling with informing their relatives, a follow up meeting can be an important way to ensure that they are provided with additional support to help them communicate. Participants also identified genetic counselors, nurses, psychologists, and general practitioners as key potential actors in the follow up process, although notably the degree to which these supporting healthcare professionals were integrated in practice varies significantly between countries. While participants supported the development of follow up procedures, they noted how the lack of dedicated resources may be an obstacle to implementation and advocated that sufficient resources be made available.

***2. Shift to a familial approach***

Throughout the workshop discussions, there were several times in which tensions arose between policies focused on individual rights compared with the perceived familial and relational nature of genetic information. Critically examining the individualistic tendencies in our ethical and legal systems, the discussions highlighted the imperative to adopt a more relational approach in clinical practice. This potentially paradigmatic shift away from an exclusive focus on individual rights and responsibilities would require a reconsideration of our current frameworks for understanding patient rights, to emphasize the inherent familial nature of genetic information and steer away from an exclusive focus on the individual.

Advocating for a broader perspective in clinical practice, the proposal discussed by participants was to treat the family as the primary unit of care, urging that ethical guidelines and policies align with this familial and relational approach. This approach has also been advocated for in the literature, most notably by Lucassen *et al.* (2007), whose joint account model acknowledges the familial aspect of genetic information obtained from an individual (index patient/proband) and underscores the possibility of disclosing such familial information without breaching patient confidentiality ^1^. However, participants noted that if the unit of care does shift beyond the patient, there arises a need for a clearer definition of "at-risk relatives."

Building on these discussion points, there was a question raised about whether existing policies like the EU General Data Protection Regulation (GDPR) could clarify the roles and responsibilities around informing at-risk relatives ^42^. The question remains whether, in the case of the processing of genetic data, there is room for a less individualistic interpretation of the regulation, whereby the “data subject” would refer not only as the patient from whom the genetic data is derived from, but whether family members could also be considered data subjects. Based on the shared nature of genetic data and the direct familial implications, family members might also be able to be considered as “data subjects” with all the rights that follow from this.

While participants generally supported the concept of shifting to a familial rather than individual approach in theory, several raised concerns about its translation into practice. Participants noted how despite the increasing traction in the ethical literature since first introduced by Lucassen *et al.* (2007) implementation has been much less straightforward in countries that have adopted familial and relational language in their guidelines and policies on genetic risk communication. For some these challenges in implementation made this shift seem like a distant possibility, with many legal reforms being required to make this reframing tenable. For other participants, there was a desire for an aspirational push to reform legal framing to indicate to patients from the outset that the use and disclosure of familial information for the care of relatives would be a possible outcome of genomic sequencing.

***3. Roles and responsibilities in the communication process***

In some circumstances it was agreed that patients are best situated to inform at-risk relatives due to their (presumably) pre-existing relationship, with one participant even noting how the act of communicating genetic risk can help strengthen or reinforce relationships. However, another central theme underpinning many of the workshop discussions was an acknowledgement of the limitations of patient led (also known as proband-mediated or family-mediated) communication. In light of these challenges, participants expressed the need to explore alternative approaches, whereby healthcare professionals adopt a more active role in the communication process, such as through the direct contact model of informing relatives ^43^. Alternatives to patient-led disclosure have been gaining traction, with countries such as the Netherlands, Switzerland, Sweden, Denmark, Finland, and Australia, investigating the acceptability of different levels of involvement of healthcare professionals in recent years ^10, 14, 44– 46^.

Although some skepticism persists regarding a shift away from a strictly patient-led approach to disclosure, a growing body of literature suggests that both patients and the public may not be averse to disclosure led by healthcare professionals or services ^13, 44– 47^. The workshop did not reach a clear consensus on the degree of involvement that healthcare professionals should have in the communication process. Opinions were particularly divided in cases where patients explicitly object to disclosure. Some participants were supportive of healthcare professionals having the ability to inform at-risk relatives (especially those from legal systems from which this is already possible) citing the potential benefit to those relatives. Others were skeptical due to concerns over the patient-clinician relationship such as how disclosing without the patient consent could negatively impact patients’ trust that healthcare professionals will prioritize their care and best interests which is central to the fiduciary relationship between patients and healthcare professionals. Furthermore, participants voiced concerns about the potential burden such responsibilities could place on healthcare professionals. Participants noted that while these challenges are significant, they must be weighed against the limitations of patient-led communication in scenarios where patients are either unwilling or unable to inform relatives themselves. It was noted that direct contact by healthcare professionals may offer a viable alternative, particularly when patients have distant or strained relationships with their relatives ^48^. Exploring whether the patient’s clinical geneticist is the best person to lead disclosure or whether there could be an added benefit to communication led by general practitioners, the patients’ treating specialist or public health authorities was identified as an area for further research.

*Exploring the role of non-genetics healthcare professionals in disclosure*

Workshop discussions raised the question of whether practitioners outside of the genetics specialty could be better situated to facilitate follow-up with patients and disclosure to their at-risk relatives. For instance, one participant suggested that primary care providers try to follow up with patients to see whether they have informed their relatives or whether they need further support. Expanded collaboration between genetics specialists and other healthcare professionals could help redistribute some of the responsibility and resource burden placed on genetics centers. Furthermore, structural patient engagement could help to develop new strategies. Several participants noted how as genetics and genomics become increasingly mainstreamed, healthcare professionals such as oncologists, cardiologists, neurologists and general practitioners have an increasingly important role in providing genetic testing. It is therefore critical that future studies explore the experiences, attitudes, and practices of other healthcare professionals outside of the genetics specialty, as the potential role in family communication of other health care profession remains underexplored. Another suggestion was that it may be advantageous to inform relatives using a third party with an established relationship with the relative. This could help to reduce the conflict that healthcare professionals might experience if they are to inform relatives, particularly if it is against patient wishes. Currently, standard practice for contacting at-risk relatives is through family letters, but informing relatives through another healthcare professional could help to link relatives to the healthcare system which can help support them to make a decision about pursuing counseling or testing.

***4. Guidelines and policy***

The workshop fostered debate on the benefits and concerns of different policy approaches to family communication of genetic risk information. While the standard approach in all countries represented in the workshop was that disclosure is only permitted with the consent of the patient, in some there was an exception to patient confidentiality (at least in its current individualistic formulation) granted when the genetic condition in question is both severe and actionable ^49^. Despite severity and actionability being commonly used criteria between various national contexts, workshop participants pointed out the challenges of defining these boundaries given the variable penetrance, and expression of genetic conditions which is inherently probabilistic. While a few participants floated the idea of having a fixed list of conditions to help decision aids regarding familial disclosure, others were quick to point out how this approach may be overly restrictive and not allow for enough leeway to consider case particulars, such as the nature of the genetic information and the familial situations. Using criteria instead (such as severity and actionability) can help allow for a wider set of factors to be taken into account, although for this proposal to be tractable in practice criteria need to be sufficiently defined. However, as a consequence of not having a fixed list, it can be difficult to determine where exactly to draw the line between when it is (and is not) acceptable to disclose genetic risk information to at-risk relatives without the consent of the patient.

While there was a lack of consensus among participants regarding which policy approach best balanced the interests of patients, at-risk relatives, and healthcare professionals, participants did agree that at the very least clear guidelines and legislation are needed to help mitigate confusion and conflict in this ethically complex situation. In countries where the legislation was ambiguous and no guidelines were present, legislation may only be applicable by analogy, meaning more than one policy approach could be possible. Participants noted that misunderstandings occurred and that healthcare professionals experienced distress over having no clear guidance on what would be considered best practice in this scenario. With no clear guidance, healthcare professionals are left to make decisions on their own, which one participant stated could cause inconsistencies in care between providers. Workshop discussions made it clear that regardless of which policy was adopted, the legal parameters as well as organizational and resource availability would need to be compatible with the approach for the change to be tenable in practice. Rather than endorsing a single policy, some participants opined that a combination of approaches may be most appropriate given the different needs and preferences of patients and the wide range of genetic conditions.

Participants in countries where a specific policy regarding this issue had been adopted were quick to point out the challenges regarding interpretation and implementation. Policies remain far from straightforward, and healthcare professionals still encounter uncertainty in how to handle ethically sensitive cases where patients are unable or unwilling to communicate with their at-risk relatives. These findings from the workshop have similarly been reflected in the literature originating from the UK, France, and Australia ^22, 33, 50, 51^. Participants seemed to agree that continual investigation and evaluation of guidelines and policies is needed to improve their implementation and best mitigate their drawbacks.

Below we present the three key policy areas discussed during the workshop:

*I. The patient’s role in disclosure*

A consensus was established early in the workshop that in some cases patients have an ethical responsibility to inform their at-risk relatives. Participants were in agreement that healthcare professionals should emphasize this responsibility to patients at various time points and proactively offer support. Where opinions diverged was on the question of whether this ethical responsibility should be backed by legislation. Many participants voiced concerns over whether under such a framework there could be any legal consequences if patients fail to inform their relatives. As of yet, in France the effects of the legislation have been seemingly less direct, with French participants noting how their national legislation helps emphasize to patients and healthcare professionals the importance of informing at-risk relatives. This sentiment has been echoed in empirical research conducted with French genetics professionals who when asked about the law’s impact on practice stated that in cases where patients appeared hesitant to disclose, the weight of the law could be a helpful tool to implore patients to inform their relatives ^22^. Despite these purported benefits, several participants remained unconvinced that the patient’s ethical duty should translate to a legal duty. These participants were concerned that if legislation results in lawsuits being filed against family members for a failure to communicate, this could be very damaging and in conflict with the initial aim to prevent harm. Another participant also cautioned against using cases of active nondisclosure, which are an outlier, as the basis for legislation stating instead that proposed policies must be proportionate and not lose sight of the much more common type of nondisclosure (passive nondisclosure) where patients do want to share relevant information with their families but may have misunderstood what to disclose or to whom the information is relevant and just need more support.

*II. Healthcare professionals’ roles and responsibilities*

While many workshop participants agreed that healthcare professionals have some kind of responsibility towards at-risk relatives, they were divided on their interpretations of what constitutes fulfillment of this duty. Some felt that it was sufficient to inform patients of the importance of communication with relatives and to provide counseling if requested; while some healthcare professionals may want to do more, these participants felt that it is important that the inherent limitations of this process are acknowledged, particularly since instances of active nondisclosure make up only a small percentage of cases.

In contrast, others were of the opinion that further action is needed to ensure that information about genetic risk is disseminated within the family. There is a need to develop a consensus on what constitutes good practice regarding the healthcare professional’s duty. Given the prevalence of (active or passive) nondisclosure and the harm that can occur as a result, it might not be sufficient to just inform patients of the importance of communication. Based on the workshop discussions, we therefore suggest that healthcare professionals encourage disclosure and follow up with patients to ensure that patients are supported throughout the communication process. If healthcare professionals suspect that patients have not informed their at-risk relatives, they should again emphasize the importance of disclosure to patients and offer genetic counseling and psychological support to encourage and support disclosure.

*III. A public health approach: the role of national registries*

Prior to the workshop, most participants were unaware that Denmark has utilized a national registry to contact relatives at risk of certain genetic conditions. This lack of awareness seemed partly due to public health discussions occurring separately from the bioethical discourse on the topic. In Denmark, the motivation for adopting this approach stems from the relatively high prevalence of conditions such as hereditary colorectal, breast, and ovarian cancers in the population, as well as the availability of risk-reducing and surveillance options. These measures aim to alleviate the burden on individuals at risk and on the healthcare system as a whole. While most participants felt that this approach would not be suitable for all genetic conditions, they did see its value in this specific context and believed this approach could serve well in conjunction with one of the other policy approaches. However, they did note that the implementation of such an approach required significant national infrastructure and public trust in the healthcare system, which in some countries seemed unlikely to be possible in the near future. Efforts would need to be made to structurally engage with patients to ultimately ensure that patients and the public are aware of the existence of the possibility of the healthcare system disclosing genetic information to family members in this particular context.

***5. Adequate resources***

Even if the ethical and legal conflicts on the issue of family communication can be resolved, significant practical challenges remain. To better support patients in the communication process, participants stated that governments must invest in both practitioner training and health infrastructure to ensure necessary resources are available. The practitioners attending the workshop stated several times how they already faced significant time and resource constraints that limit the length and depth of consultation with patients. Currently some healthcare professionals do not have the time available to address familial implications prior to testing. Following testing, healthcare professionals again do not always have sufficient time to address these issues during the post-test consultation, and there is no current systematic support for follow up with patients about family communication. When genetic testing is introduced more broadly in the healthcare system, non-genetic healthcare professionals need education in genetics and either time to support patients in family communication or resources for referral to a clinical genetic unit for counseling of the patient, including support in risk disclosure to at-risk relatives. Without adequate competence, or time, healthcare professionals cannot be expected to sufficiently address results with familial implications and the importance of family communication. Therefore, structural support to help alleviate restraints on healthcare professionals could be beneficial. Clear guidance (such as by law or professional guidelines) for various healthcare providers offering genomic information will allow proportional resource commitment and optimized, efficient care, communication and support for patients and their families.

Additionally, further integrating other healthcare professionals and supporting staff, such as genetic counselors and psychologists, was identified as a crucial strategy to better support patients as they navigate the complex communication process. Genetic counselors and psychologists can play a crucial role as mediators between patients and clinical geneticists. One participant reported how stigma and practical burdens of pursuing additional care may deter some patients from utilizing counseling and psychological support. They suggested therefore that these professionals should be integrated in the consultation sessions as early as possible to reduce the likelihood of this occurring. Additional resources are also needed to help educate healthcare professionals outside of the genetics specialty in how to address family communication.

E-Health solutions emerged as a potential avenue for further exploration. Proposed strategies included the creation of materials to guide patients on how to inform at-risk family members and foster conversations. More specifically, the creation of tools to assist patients in communicating with their family members, such as role play videos and a set of versatile 'tell the family tools' (with several modules for different patients, family members, genetic conditions, and contexts), were suggested as a means to cater to diverse patient needs. These could be made available to patients through an online platform. Ensuring equitable access to these tools would be necessary, particularly in this context for individuals who may face challenges in accessing digital modules ^52^.

## Conclusion

Given the familial implications of genomic data, it is imperative to strike a balance between the rights and responsibilities of patients, at-risk relatives, and healthcare professionals. Based on our workshop discussions with interdisciplinary and international experts, we present several challenges stemming from the intricate process of family communication of genetic risk information, which will become more acute as the mainstreaming of genomics advances. Developing adequate guidance—in the form of not just guidelines and policies, but also through education and engagement—for healthcare professionals and patients is crucial to better navigate this process. Enhancing pre-test counseling and follow up procedures, implementing policies to clarify roles and responsibilities, and training healthcare professionals (both within and outside genetics services) are essential steps to address concerns related to communicating genetic risk in families.

## Data Availability

No data are associated with this article.

## References

[1] Lucassen A : Should families own genetic information? Yes. *BMJ.* 2007;335(7609):22. 10.1136/bmj.39252.386030.AD 17615220 PMC1910686

[2] Gaff CL Clarke AJ Atkinson P : Process and outcome in communication of genetic information within families: a systematic review. *Eur J Hum Genet.* 2007;15(10):999–1011. 10.1038/sj.ejhg.5201883 17609674

[3] Derbez B : Is there a "right time" for bad news? Kairos in familial communication on hereditary breast and ovarian cancer risk. *Soc Sci Med.* 2018;202:13–19. 10.1016/j.socscimed.2018.02.022 29500986

[4] Black L McClellan KA Avard D : Intrafamilial disclosure of risk for hereditary breast and ovarian cancer: points to consider. *J Community Genet.* 2013;4(2):203–14. 10.1007/s12687-012-0132-y 23275181 PMC3666841

[5] Clarke A Richards M Kerzin-Storrar L : Genetic professionals' reports of nondisclosure of genetic risk information within families. *Eur J Hum Genet.* 2005;13(5):556–62. 10.1038/sj.ejhg.5201394 15770225

[6] Geelen E Van Hoyweghen I Horstman K : Making genetics not so important: family work in dealing with familial hypertrophic cardiomyopathy. *Soc Sci Med.* 2011;72(11):1752–9. 10.1016/j.socscimed.2010.06.012 20630643

[7] Suthers GK Armstrong J McCormack J : Letting the family know: balancing ethics and effectiveness when notifying relatives about genetic testing for a familial disorder. *J Med Genet.* 2006;43(8):665–70. 10.1136/jmg.2005.039172 16371501 PMC2564590

[8] van den Heuvel LM Smets EMA van Tintelen JP : How to inform relatives at risk of hereditary diseases? A mixed-methods systematic review on patient attitudes. *J Genet Couns.* 2019;28(5):1042–1058. 10.1002/jgc4.1143 31216099

[9] Menko FH Aalfs CM Henneman L : Informing family members of individuals with Lynch syndrome: a guideline for clinical geneticists. *Fam Cancer.* 2013;12(2):319–24. 10.1007/s10689-013-9636-9 23535968

[10] Pedrazzani C Aceti M Schweighoffer R : The communication chain of genetic risk: analyses of narrative data exploring proband-provider and proband-family communication in hereditary breast and ovarian cancer. *J Pers Med.* 2022;12(8):1249. 10.3390/jpm12081249 36013197 PMC9409642

[11] Daly MB Montgomery S Bingler R : Communicating genetic test results within the family: is it lost in translation? A survey of relatives in the randomized six-step study. *Fam Cancer.* 2016;15(4):697–706. 10.1007/s10689-016-9889-1 26897130 PMC5010833

[12] Ahsan MD Levi SR Webster EM : Do people with hereditary cancer syndromes inform their at-risk relatives? A systematic review and meta-analysis. *PEC Innov.* 2023;2: 100138. 10.1016/j.pecinn.2023.100138 37214514 PMC10194207

[13] Öfverholm A Karlsson P Rosén A : The experience of receiving a letter from a cancer genetics clinic about risk for hereditary cancer. *Eur J Hum Genet.* 2024;32(5):539–544. 10.1038/s41431-024-01551-9 38355958 PMC11061288

[14] Menko FH van der Velden SL Griffioen DN : Does a proactive procedure lead to a higher uptake of predictive testing in families with a pathogenic / variant? A family cancer clinic evaluation. *J Genet Couns.* 2024;33(3):615–622. 10.1002/jgc4.1767 37605508

[15] Lindberg LJ Wadt KAW Therkildsen C : National experiences from 30 years of provider-mediated cascade testing in lynch syndrome families-the Danish model. *Cancers (Basel).* 2024;16(8):1577. 10.3390/cancers16081577 38672659 PMC11048852

[16] Ballard LM Band R Lucassen AM : Interventions to support patients with sharing genetic test results with at-risk relatives: a Synthesis Without Meta-analysis (SWiM). *Eur J Hum Genet.* 2023;31(9):988–1002. 10.1038/s41431-023-01400-1 37344572 PMC10474271

[17] Paneque M Shea RO Narravula A : Thirty-years of genetic counselling education in Europe: a growing professional area. *Eur J Hum Genet.* 2024;32(11):1500–1505. 10.1038/s41431-024-01552-8 38355960 PMC11576746

[18] Abacan M Alsubaie L Barlow-Stewart K : The global state of the genetic counseling profession. *Eur J Hum Genet.* 2019;27(2):183–197. 10.1038/s41431-018-0252-x 30291341 PMC6336871

[19] Mendes A Paneque M Sousa L : How communication of genetic information within the family is addressed in genetic counselling: a systematic review of research evidence. *Eur J Hum Genet.* 2016;24(3):315–25. 10.1038/ejhg.2015.174 26264439 PMC4755382

[20] Dheensa S Lucassen A Fenwick A : Limitations and pitfalls of using family letters to communicate genetic risk: a qualitative study with patients and healthcare professionals. *J Genet Couns.* 2018;27(3):689–701. 10.1007/s10897-017-0164-x 29094272 PMC5943374

[21] Phillips A Vears DF Van Hoyweghen I : Clinician perspectives on policy approaches to genetic risk disclosure in families. *Fam Cancer.* 2024;23(2):177–186. 10.1007/s10689-024-00375-2 38548926 PMC11233314

[22] Audiffret Van Haecke DD de Montgolfier S : Genetic test results and disclosure to family members: qualitative interviews of healthcare professionals' perceptions of ethical and professional issues in France. *J Genet Couns.* 2016;25(3):483–94. 10.1007/s10897-015-9896-7 26482743

[23] Derbez B de Pauw A Stoppa-Lyonnet D : Familial disclosure by genetic healthcare professionals: a useful but sparingly used legal provision in France. *J Med Ethics.* 2019;45(12):811–816. 10.1136/medethics-2018-105212 31462451

[24] Parker M Lucassen AM : Genetic information: a joint account? *BMJ.* 2004;329(7458):165–7. 10.1136/bmj.329.7458.165 15258076 PMC478234

[25] Lucassen A Gilbar R : Alerting relatives about heritable risks: the limits of confidentiality. *BMJ.* 2018;361:k1409. 10.1136/bmj.k1409 29622529 PMC5885756

[26] Gilbar R Barnoy S : Facing legal barriers regarding disclosure of genetic information to relatives. *New Genetics and Society.* 2020;39(4):483–501. 10.1080/14636778.2020.1755639

[27] Brownsword R Wale J : The right to know and the right not to know revisited: part one. *Asian Bioeth Rev.* 2017;9(1):3–18. 10.1007/s41649-017-0012-1 28943969 PMC5585997

[28] Mendes Á, Paneque M Clarke A : Choosing not to know: accounts of non-engagement with pre-symptomatic testing for Machado-Joseph disease. *Eur J Hum Genet.* 2019;27(3):353–9. 10.1038/s41431-018-0308-y 30573801 PMC6460576

[29] Chadwick R Levitt M Shickle D : The right to know and the right not to know: genetic privacy and responsibility.2 ed. Cambridge: Cambridge University Press;2014. Reference Source

[30] Otlowski M : Australian reforms enabling disclosure of genetic information to genetic relatives by health practitioners. *J Law Med.* 2013;21(1):217–34. 24218794

[31] Otlowski MF : Disclosing genetic information to at-risk relatives: new Australian privacy principles, but uniformity still elusive. *Med J Aust.* 2015;202(6):335–7. 10.5694/mja14.00670 25832164

[32] Tiller J Bilkey G Macintosh R : Disclosing genetic information to family members without consent: five Australian case studies. *Eur J Med Genet.* 2020;63(11): 104035. 10.1016/j.ejmg.2020.104035 32805446

[33] Meggiolaro N Barlow-Stewart K Dunlop K : Disclosure to genetic relatives without consent – Australian genetic professionals’ awareness of the health privacy law. *BMC Med Ethics.* 2020;21(1): 13. 10.1186/s12910-020-0451-1 32019532 PMC7001268

[34] National Health and Medical Research Council AG: Use and disclosure of genetic information to a patient's genetic relatives under section 95AA of the Privacy Act 1988 (Cth). National Health and Medical Research Council;2024. Reference Source

[35] Anna M Christine P Jonathan R : Professional duties are now considered legal duties of care within genomic medicine. *Eur J Hum Genet.* 2020;28(10):1301–4. 10.1038/s41431-020-0663-3 32514131 PMC7609317

[36] Rothstein MA : Reconsidering the duty to warn genetically at-risk relatives. *Genet Med.* 2018;20(3):285–90. 10.1038/gim.2017.257 29388945

[37] LOI n° 2021-1017 du 2 août 2021 relative à la bioéthique. 2021. Reference Source

[38] Phillips A Bronselaer T Borry P : Informing relatives of their genetic risk: an examination of the Belgian legal context. *Eur J Hum Genet.* 2022;30(7):766–71. 10.1038/s41431-021-01016-3 34997232 PMC9259709

[39] Pastorino R Pezzullo AM Osti T : The PROPHET project paves the way for personalized prevention in the future healthcare. *Eur J Cancer Prev.* 2024;33(5):387–9. 10.1097/CEJ.0000000000000873 38598497

[40] Young AL Butow PN Tucker KM : Challenges and strategies proposed by genetic health professionals to assist with family communication. *Eur J Hum Genet.* 2019;27(11):1630–8. 10.1038/s41431-019-0447-9 31189929 PMC6871519

[41] Li ST Sun S Lie D : Factors influencing the decision to share cancer genetic results among family members: an in-depth interview study of women in an Asian setting. *Psychooncology.* 2018;27(3):998–1004. 10.1002/pon.4627 29314485

[42] Regulation (EU) 2016/679 of the European Parliament and of the Council of 27 April 2016 on the protection of natural persons with regard to the processing of personal data and on the free movement of such data, and repealing Directive 95/46/EC (General Data Protection Regulation).2016. Reference Source

[43] Schwiter R Rahm AK Williams JL : How can we reach at-risk relatives? Efforts to enhance communication and cascade testing uptake: a mini-review. *Current Genetic Medicine Reports.* 2018;6(2):21–7. 10.1007/s40142-018-0134-0

[44] Andersson A Hawranek C Öfverholm A : Public support for healthcare-mediated disclosure of hereditary cancer risk information: results from a population-based survey in Sweden. *Hered Cancer Clin Pract.* 2020;18: 18. 10.1186/s13053-020-00151-0 PMC7493346 32944097

[45] Hawranek C Hajdarevic S Rosén A : A focus group study of perceptions of genetic risk disclosure in members of the public in Sweden: "I'll phone the five closest ones, but what happens to the other ten? *J Pers Med.* 2021;11(11): 1191. 10.3390/jpm11111191 34834542 PMC8622605

[46] Petersen HV Frederiksen BL Lautrup CK : Unsolicited information letters to increase awareness of Lynch syndrome and familial colorectal cancer: reactions and attitudes. *Fam Cancer.* 2019;18(1):43–51. 10.1007/s10689-018-0083-5 29651783

[47] Newson AJ Humphries SE : Cascade testing in familial hypercholesterolaemia: how should family members be contacted? *Eur J Hum Genet.* 2005;13(4):401–8. 10.1038/sj.ejhg.5201360 15657617

[48] Nääs C von Salomé J Rosén A : Patients’ perceptions and practices of informing relatives: a qualitative study within a randomised trial on healthcare-assisted risk disclosure. *Eur J Hum Genet.* 2024;32(4):448–55. 10.1038/s41431-024-01544-8 38308085 PMC10999412

[49] Phillips A Borry P Van Hoyweghen I : Disclosure of genetic information to family members: a systematic review of normative documents. *Genet Med.* 2021;23(11):2038–46. 10.1038/s41436-021-01248-0 34234303

[50] d'Audiffret Van Haecke D de Montgolfier S : Genetic diseases and information to relatives: practical and ethical issues for professionals after introduction of a legal framework in France. *Eur J Hum Genet.* 2018;26(6):786–95. 10.1038/s41431-018-0103-9 29487415 PMC5974143

[51] Dheensa S Fenwick A Lucassen A : Approaching confidentiality at a familial level in genomic medicine: a focus group study with healthcare professionals. *BMJ Open.* 2017;7(2): e012443. 10.1136/bmjopen-2016-012443 28159847 PMC5293977

[52] Phillips A Vears DF Hoyweghen IV : Digital tools for sharing genetic information with family members. *Lancet Oncol.* 2020;21:891–2. Reference Source

