# Supplementary material for: Genomic findings with familial implications: agenda setting in light of mainstreaming
Source: Open Res Eur. 2025 Jan 10;5:4. [Version 1] doi: 10.12688/openreseurope.19128.1 (PMC11982804; doi:10.12688/openreseurope.19128.1)
Supplement: Peer review history

# Reviewer report: Reviewer response for version 1

**DOI:** 10.21956/openreseurope.20699.r52166

**Published:** 2025-04-09

**Authors:** Anna Plym

**Affiliations:** 1 Karolinska Institutet, Stockholm, Sweden

This article provides a timely summary of an international workshop addressing communication of genetic test results that may have implications for family members. The authors identified five key themes from the discussions and highlighted the need of developing guidelines that can help communication but also overall navigation in this complex process.

 The authors have provided a rich discussion on different aspects of communication of individual genetic test results to family members that may be affected. This includes the role of the individual and the role of the health care system (whose responsibility is it to communicate?), challenges related to an individual’s own struggle in coping with a severe diagnosis at the same time, distinguishing between transmitting information vs. engaged communication (the role of pre- and post-test counselling), and communication around what being at elevated genetic risk means (addressing uncertainty and probabilities but also severity and actionability).

 I fully agree with the authors' conclusions that the development of clear guidelines in this area is essential, also in the light of the expected increase in the use of genetic testing.

 I have no concerns or additional comments to this well-written article other than to emphasise the need of rigorous research studies that can help understand what it means to be an at-risk relative, in particular in terms of absolute lifetime disease risk and the influence of potential risk mitigation approaches. Any communication without such information will be inherently difficult.

Where applicable, are recommendations and next steps explained clearly for others to follow? (Please consider whether others in the research community would be able to implement guidelines or recommendations and/or constructively engage in the debate)

Yes

Does the article adequately reference differing views and opinions?

Yes

Are all factual statements correct, and are statements and arguments made adequately supported by citations?

Yes

Is the rationale for the Open Letter provided in sufficient detail? (Please consider whether existing challenges in the field are outlined clearly and whether the purpose of the letter is explained)

Yes

Is the Open Letter written in accessible language? (Please consider whether all subject-specific terms, concepts and abbreviations are explained)

Yes

Reviewer Expertise:

Genetic epidemiology, rare and common inherited genetic variation, prostate cancer risk

I confirm that I have read this submission and believe that I have an appropriate level of expertise to confirm that it is of an acceptable scientific standard.

# Reviewer report: Reviewer response for version 1

**DOI:** 10.21956/openreseurope.20699.r49959

**Published:** 2025-02-07

**Authors:** Mutaz Amin

**Affiliations:** 1 Al-Neelain University, Khartoum, Sudan

In the article entitled: "Genomic findings with familial implications: agenda setting in light of mainstreaming", the authors address the challenges associated with informing family members with the potential risks of genetic testing results. The article is the outcome of a workshop conducted in Belgium. The article is clear and very well-written and is an important addition to the literature addressing a very complicated but need to be discussed issue. I have no reservations, comments or suggestions.

Where applicable, are recommendations and next steps explained clearly for others to follow? (Please consider whether others in the research community would be able to implement guidelines or recommendations and/or constructively engage in the debate)

Yes

Does the article adequately reference differing views and opinions?

Yes

Are all factual statements correct, and are statements and arguments made adequately supported by citations?

Yes

Is the rationale for the Open Letter provided in sufficient detail? (Please consider whether existing challenges in the field are outlined clearly and whether the purpose of the letter is explained)

Yes

Is the Open Letter written in accessible language? (Please consider whether all subject-specific terms, concepts and abbreviations are explained)

Yes

Reviewer Expertise:

Rare disease genetics and genomics

I confirm that I have read this submission and believe that I have an appropriate level of expertise to confirm that it is of an acceptable scientific standard.
